# Anticancer perspective of 6-shogaol: anticancer properties, mechanism of action, synergism and delivery system

**DOI:** 10.1186/s13020-023-00839-0

**Published:** 2023-10-24

**Authors:** Yaoxia Jia, Xing Li, Xiangqi Meng, Jinjie Lei, Yangmiao Xia, Lingying Yu

**Affiliations:** 1 College of Pharmacy, Chengdu University of Traditional Chinese Medicine, No. 1166 Liutai Avenue, Chengdu, 611137 China; 2 Jianyang Chinese Medicine Hospital, Chengdu, 641400 China; 3 State Key Laboratory of Southwestern Chinese Medicine Resources, Chengdu, China

**Keywords:** Ginger, 6-Shogaol, Anti-cancer, Mechanism, Toxicity, Synergy, Delivery system

## Abstract

Cancer is a malignant disease that has plagued human beings all the time, but the treatment effect of commonly used anticancer drugs in clinical practice is not ideal by reason of their drug tolerance and Strong adverse reactions to patients. Therefore, it is imperative to find effective and low-toxic anticancer drugs. Many research works have shown that natural products in Chinese herbal medicine have great anticancer potential, such as 6-shogaol, a monomer composition obtained from Chinese herbal ginger, which has been confirmed by numerous in vitro or vivo studies to be an excellent anti-cancer active substance. In addition, most notably, 6-shogaol has different selectivity for normal and cancer cells during treatment, which makes it valuable for further research and clinical development. Therefore, this review focus on the anti-cancer attributes, the mechanism and the regulation of related signaling pathways of 6-shogaol. In addition, its synergy with commonly used anticancer drugs, potential drug delivery systems and prospects for future research are discussed. This is the first review to comprehensively summarize the anti-cancer mechanism of 6-shogaol, hoping to provide a theoretical basis and guiding significance for future anti-cancer research and clinical development of 6-shogaol.

**Graphical Abstract:**

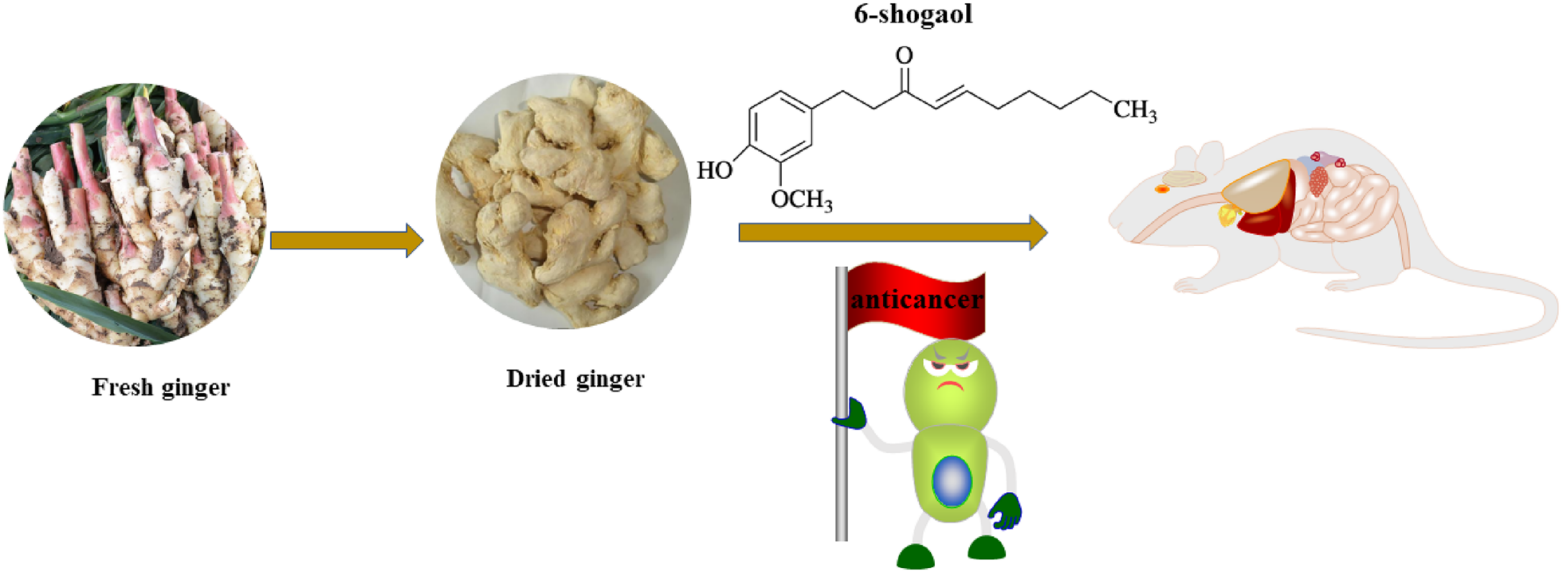

## Introduction

Cancer is the main reason of human mortality and one of the most difficult hurdles for every country in the world to overcome to increase the life expectancy of its population [1]. Chinese herbal medicine has excellent anticancer and anti-inflammatory activities, rich in anticancer compounds, and has direct toxic and indirect regulatory effects on the tumor microenvironment and cancer cells [2]. Chinese herbal medicine has been widely used in clinical practice, which can enhance the body's immunity, improve patients' quality of life, and reduce adverse reactions [3]. Therefore, traditional Chinese medicine is receiving increasing attention from people. Ginger (Zingiber officinale Roscoe) is the rhizome of plants in the ginger family and the most common herb in traditional Chinese medicine. Due to its aromatic and pungent taste, it is often used as a spice and seasoning in various foods and beverages [4]. It is an important component of various folk medicine systems around the world, used to treat various diseases, such as colds, headaches, nausea, stomach diseases, diarrhea, inflammation, and rheumatism, or as a wind repellent, anti-bloating agent, and digestive agent [5]. In addition, ginger is used as a plant supplement therapy, showing anti-inflammatory, antioxidant and anti-cancer effects, as well as nausea and vomiting caused by chemotherapy for breast cancer [6]. Ginger has been proven effective in treating cancer, and in Singapore, cooked ginger rhizomes are used as a means of preventing cancer [7]. An infusion of the rhizome is used against breast cancer by the Palestinians [8]. Soup prepared from ginger root, turmeric, and honey as a commonly used treatment for general cancer [9]. Another recipe used by the Palestinians to manage stomach and liver cancer uses 100 g of the ground dried rhizomes boiled in water and given twice daily after meals. The nutritional and health value and medicinal function of ginger mainly stem from its bioactive components, especially the pungent component gingerols and its dehydrated product shogaols. Research has found that shogaols have stronger biological activity than gingerols, especially 6-shogaol [10].

**Fig. 1:**
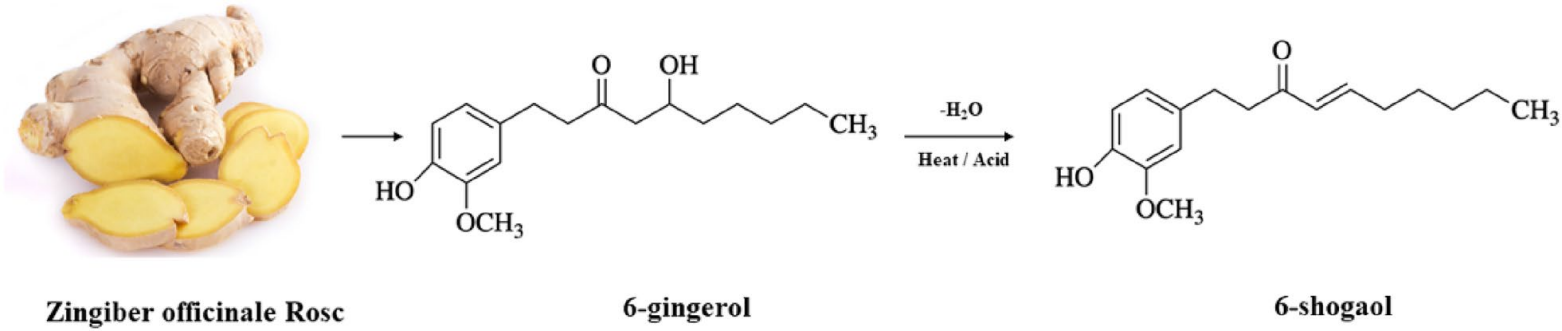
6-Shogaol production pathway

6-shogaol is formed by dehydration of 6-gingerol from the Chinese herb ginger (*Zingiber officinale Rosc*) (Fig. 1) [10], with anti-cancer, anti-inflammatory and anti-oxidant biological activities [11]. It has been found that 6-shogaol has an advantage over 6-gingerol in terms of anticancer, antioxidant and anti-inflammatory effects, which may be attributed to the chemical structure of 6-shogaol, which contains an α,β-unsaturated carbonyl group (Michael receptor) [4]. 6-shogaol has been used in a variety of diseases, including cancer [10]. The anticancer efficacy of 6-shogaol has been confirmed and recognized in many cancer models, such as breast [12], cervical [13], colon [14], liver [15], kidney [16], oral [17], and prostate cancer [18].

## Discovery, synthesis and metabolism of 6-shogaol

### History of the discovery

The pungent compound 6-shogaol was identified and first described by Nomura in 1918 [4]. In 1928, Nomura and Iwamoto demonstrated the existence of hydroxyl groups in ginger phenolic compounds through the Zerevitinov method. In 1929, Nomura, Iwamoto, and Murakami confirmed the conjugated structure of the 6-shogaol unsaturated carbonyl system. Subsequently, Nomura and Tsurumi suggested the structural formula of “4-hydroxy-3-methoxyphenylethyl n-heptenyl ketone” for shogaol and synthesized the compound for the first time [10].

### Chemical synthesis

6-shogaol is prepared by dehydration of 6-gingerol under acidic or thermally catalyzed conditions, as β- Hydroxyl ketone functional groups are easily formed during the heating process α, β- Unsaturated ketone [19]. HCL or p-toluenesulfonic acid are commonly used as acidic catalysts, however, these reagents may lead to environmental pollution, so the following environmentally friendly and convenient methods for the synthetic preparation of 6-shogaol have been investigated, such as vapour processing [20], microwave processing [21] and Ultrasound-assisted catalytic dehydration of acidic ionic liquids [22]. Among them, ultrasound-assisted acidic ionic liquid catalysis has the advantages of convenience, environmental protection and high efficiency, and the yield of 6-shogaol in this method can reach up to 97.16%. In this method, 1-butyl-3-methylimidazolium hydrosulfate ionic liquid ([Bmin]HSO_4_) is used as a catalyst, which is stable and non-toxic. The optimum conditions were 2.5:10 mass ratio of [Bmim]HSO_4_ to ginger oleoresin, ultrasonic power of 300 W, reaction temperature of 80 °C and reaction time of 30 min [22].

### Metabolism in the body

When administered orally, 6-gingerol undergoes severe first pass metabolism in the gastrointestinal tract and liver, with only trace amounts of 6-shogaol entering the bloodstream in free form [10]. The mercapturic acid pathway is the main metabolic pathway of 6-shogaol [23], So far, at least 28 metabolites of 6-shogaol have been identified from mouse feces and urine [24]. In the gastrointestinal tract, 6-shogaol is converted under the influence of the intestinal microbiota to two major metabolites, 1-(4′-hydroxy-3′-methoxyphenyl)-decan-3-ol (M9) and 1-(4′-hydroxy-3′-methoxyphenyl)-decan-3-one (M11) [25]. In animal liver, the metabolites of 6-shogaol mainly contain two oxidative metabolites (1E,4E)-1-(4′-hydroxy-3′-methoxyphenyl)-deca-1,4-dien-3-one (6-dehydroshogaol) and (E)-1-(4′-hydroxy -3′-methoxyphenyl)-dec-1-en-3-one (6-dehydroparadol) as well as three reductive metabolites, 1-(4′-hydroxy-3′-methoxyphenyl)-decan-3-one (6-paradol), 1-(4′- hydroxy-3′-methoxy)-decan-3-ol (M9) and 1-(4′-hydroxy-3′-methoxyphenyl)-deca-4-ene-3-ol (M6) [26]. In addition, target tissues (such as cancer cells) are also important sites for the biological transformation of 6-shogaol. After incubation with HCT-116 human colon cancer cells for 24 h, the main metabolites produced are M6, M9, 6-paramol, and 5-glutathiol-6-shogaol (M13). In HT-29 human colon cancer cells, H-1299 human lung cancer cells, and CL-13 mouse lung cancer cells, M9 and M13 are the main metabolites [27]. It is worth noting that some metabolites have stronger biological activity than 6-shogaol [28].

## Anti-cancer properties of 6-shogaol

### Toxicity specificity

Many in vitro and in vivo studies have confirmed that 6-shogaol exhibits no or little toxic effects on normal cells/tissues, while its dose can significantly kill cancer cells. In breast cancer cells, 6-shogaol effectively kills breast cancer stem cells, including monolayers and spheroids, at a dose that is non-toxic to non-cancerous cells [12]. In liposarcoma cell lines, 6-shogaol can significantly inhibit the growth of SW872 and 93T449 cells without affecting the growth of normal 3T3-L1 adipocytes [29]. In the colorectal cancer model, 6-shogaol at a concentration of 80 μM showed high toxicity to human colon cancer cells SW480 and SW620 at 95 and 90%, respectively, whereas the viability of normal fibroblasts WI38 was only reduced by 17% [30]. In acute leukemia models, 10 and 20 μM of 6-shogaol induced significant apoptosis in primary leukemic monocytes while having no or almost no effect on apoptosis of normal bone marrow monocytes. In addition, the investigators conducted in vivo studies in mice on this basis. In vivo research results indicated that 6-shogaol significantly inhibited tumor growth in U937 (human histiocytic lymphoma) xenografts without causing side effects in mice [31]. The same conclusion was obtained in the liver cancer xenograft model mice, where 6-shogaol can significantly interfere with the growth of xenograft tumors without other toxic and side effects in the mice [32].

In prostate cancer, 6-shogaol can significantly diminish the volume and weight of tumors at a dose of 100 mg/kg in mice, however, 6-shogaol-treated mice did not cause other significant changes, such as body weight or daily food consumption, compared to control mice. Furthermore, the researchers found that normal tissues showed no abnormalities by performing necropsy studies on 6-shogaol-treated mice [33]. It is thought that the selectivity of action of 6-shogaol on normal and tumor cell lines may be related to the increased ROS production induced by 6-shogaol, but the specific reason is still unknown so far [34]. Based on in vitro and in vivo studies, 6-shogaol not only has a good anti-cancer effect but also has a different sensitivity to cancer and noncancer cells. This anti-cancer characteristic makes it valuable to be studied in depth.

### In vitro anticancer activity

It has been confirmed by numerous experimental studies that 6-shogaol exhibits strong anti-proliferative activity against various types of tumors, including breast, liver, cervical, oral, colon, and renal cancers, as shown in Table 1. Concentrations of 6-shogaol in general in vitro anticancer studies range from 0 to 240 μM. In this concentration range, the potency of 6-shogaol to inhibit the proliferation of cancer cells was positively correlated with the dose and time of administration, while not affecting normal cells. Notably, in a breast cancer model, T47D cells showed high sensitivity to 6-shogaol (IC_50_ = 0.5 ± 0.1), which showed comparable cytotoxicity to the chemotherapeutic agent cisplatin (IC_50_ = 0.7 ± 0.2 μM) [35]. In addition, 6-shogaol was found to have high inhibitory activity against breast cancer spheroid cells, especially against MDA-MB-231 spheroid cells, through comparative anticancer efficacy studies with paclitaxel, a drug commonly used in breast cancer, whereas paclitaxel, although showing strong activity in monolayers, did not show activity against spheroids even at concentrations 10,000 times higher than 6-shogaol [12]. This indicates that 6-shogaol has great therapeutic potential for the therapy of breast cancer.

**Table 1:** 6-Shogaol in vitro anticancer study (“↑” represents increase/promotion/activation while “↓” represents inhibition/reduction/inactivation. “-”Indicates not indicated.)

Cancer models	Cell lines	Mechanisms	Concentration used	IC_50_	Ref.
Breast cancer	MCF-7 MDA-MB-231	Induce G2/M phase cell cycle arrest Induce apoptosis: Notch1↓ Hes↓ cyclinD1↓ Induce cell autophagy	1–100 μM	MCF-7 7.94 ± 0.57 μM MDA-MB-231 5.67 ± 0.73 μM	[12]
	T47D	Induce cell autophagy Induce apoptosis Induce G2/M phase cell cyclearrest: Notch↓ Hes1↓ CyclinD1↓	0–100 μM	0.5 ± 0.1 μM	[35]
	MCF-7 MDA-MB-231	Inhibit cancer cell invasion Inhibit NF-kB cascade activation: MMP-9↓ p-JNK↓ p–c-jun↓ p65↓	5–30 μM	_	[36]
	MDA-MB-231	Induce ER stress: polyubiquitinated proteins↑ Bip↑ CHOP↑ Induce G1 phase cell cycle arrest	0–50 μM	22.1 ± 0.4 μM	[37]
Liver cancer	SMMC-7721	Induce apoptosis: p-elF2α↓ CHOP↑	0–20 μM	_	[38]
	HepG2 Huh-7	Induce G2/M phase cell cycle arrest: CyclinA↓、CyclinB1↓、CyclinD1↓ CyclinE1↓ cdc2↓ cdc25↓ Induce apoptosis: Caspase-3↑ ROS↑ ERK↓ p38↓ JNK↓ Induce autophagy: ER stress ROS↑ LC-3II↑ GRP78↑ p-eIF2aα↑ p-Akt↓ p-AMPK↓	10–60 μM	HepG2 50 μM Huh-7 60 μM	[15]
	HepG2 Li-7	Induce apoptosis: MRP1↓ Induce G0/G1 cell cycle arrest: AKT/mTOR/MRP1↓ CyclinA2↓ Cyclin D1↓ Cyclin E1↓	10–50 μM	_	[39]
	Hep3B	Inhibit invasion and metastasis: MAPK↓ PI3k/Akt↓ NF-kB↓ STAT3↓	10–50 μM	_	[40]
Cervical cancer	HeLa SiHa	Induce apoptosis: p-PI3K↓ p-Akt↓ p-mTOR↓ Induce G2/M phase arrest Inhibit metastasis: N-cadherin↓ Snail↓ Twist↓ Zeb-1↓ Zeb-2↓	0–160 μM	HeLa 25.68 ± 0.47 μM SiHa 37.52 ± 1.56 μM	[13]
	HeLa	Induce apoptosis: ER stress、Bax↑ Caspase-3↑ PARP↓ Induce G2/M phase arrest	5–80 μM	14.75 ± 0.94 μM	[41]
Colon cancer	SW480 SW620	Induce apoptosis Induce G2/M phase arrest	0–80 μM	20 μM	[30]
	HT-29	Induce G2/M phase arrest Induce apoptosis: survivin↓ Bcl-2↓ Bax↑	0–80 μM	40 μM	[42]
	HT-29 HCT116	Induce apoptosis: PPARγ↑ NFκB↓	0–100 μM	_	[43]
Oral cancer	YD-10B Ca9-22	Induce apoptosis: p-PI3K↓ p-AKT↓ p-mTOR↓ p53↓ caspase-3↓ Bcl-2↓ Bax↑ Inhibit cell proliferation: PI3K/AKT↓	0–20 μM	_	[44]
Kidney cancer	786-O	Inhibit PhIP-mediated osteoclastogenesis and bone resorption: IL-8↓ PTHrP↓ RANKL↓	2 mM	_	[16]
	Caki	Induce apoptosis: MMP↓ c-FLIP(L) ↓ ROS↑	0–20 μM	_	[45]
Non-small cell lung cancer	A549	Induce cell death via pyroptosis	0–50 μM	29.6 ± 2.1 μM	[37]
	A549	Induce autophagy: AKT↓ mTOR↓ GSK-3β↓ FKHR↓	0–50 μM	_	[46]
	A549	Induce apoptosis: GSH↑ p53↑ PUMA↑ Bcl-2↓ Bcl-XL↓	0–80 μM	25.17 μM	[47]
	A549	Induce apoptosis: GSK3β / β-catenin↓ mPGES-1↓	25–240 μM	_	[48]
	NCI-H1650 NCI-H520 NCI-H1975	Induce G1 or G2/M phase arrest Induce apoptosis: caspase-3↑ caspase-7↑ AKT↓ p-STAT3↓	0–20 μM	_	[49]
	H-1299	Induce apoptosis	0–80 μM	25.8 μM	[50]
Prostate cancer	LNCaP DU145 PC3	Induce apoptosis: STAT3↓ NF-KB↓ cyclin D1↓ survivin↓ cMyc↓ Bcl-2↓ Bax↑ p21↑ p27↑ SOCS1↑ IRF1↑ Anti-inflammatory: IL-7↓ CCL5↓	0–40 μM	_	[33]
	PC-3	Induce apoptosis: COX-2↓ MAPK↓ PI3K/Akt↓ cyclin D1↓ MMP-9↓ caspase↓	0–100 μM	_	[18]
Leukaemia	Nalm-6	Induce apoptosis: p53↑ p21↑ PUMA↑ FSAN↓ ROS↑	0–200 μM	191.33 ± 2.96 μM	[51]
Pancreatic cancer	BxPC-3	Induce apoptosis: TLR4 / NF-κB↓ cIAP-1↓ survivin↓ XIAP↓ Bcl-2↓ caspase 3↑ COX-2↓ cyclin D1↓PARP↑	0–30 μM	6.60 μM	[52]
Human fibrosarcoma	HT1080 cell	Induce apoptosis: ROS↑ p-mTOR↑ p-Akt↑	2.5–150 μM	52.8 μM	[34]
Melanoma	B16F10 mouse melanoma cells	Inhibit melanin synthesis: ERK↑ Tyrosinase↓	1–10 μM	_	[53]

### In vivo anticancer activity

6-shogaol shows great potential for in vitro anticancer activity, and the next step is to determine whether the in vitro anticancer results can be replicated in vivo in multiple cancer types. Due to the fact that the human body is a complex multicellular organism, in vitro results can only serve as an indicator of 6-shogaol’s anticancer activity, and only in vivo validation can confirm its anticancer efficacy. Kim et al. tested the effect of 6-shogaol on NCI-H1650 lung cancer cells using a mouse model [54]. In this study, the average tumor volume of the carrier treatment group was 393.4 mm^3^, while the average tumor volumes of mice treated with 6-shogaol at doses of 10 and 40 mg/kg were 274.7 and 140.8 mm^3^, respectively. And it was found that 6-shogaol did not cause any changes in body weight after treating tumor mice, indicating that 6-shogaol not only had good anticancer effects but also did not cause toxic side effects on mice. In FVB/N male mice injected subcutaneously with prostate cancer HMVP2 cells, the cancer cells were grown in mice for 2 weeks and then treated with 6-shogaol at a dose of 100 mg/kg, which resulted in a 48% reduction in tumour weight, a statistically significant reduction in tumour weight after treatment with 6-shogaol when compared to the control group [33]. Human colon tumor cells were implanted into female thymic nude mice. The first day and the following day after cancer cell implantation, a dose of 15 mg/kg 6-shogaol was injected intraperitoneally for intervention. After 30 days of treatment, although the tumor was not completely eliminated, it significantly inhibited the growth of tumor cells [55]. 6-shogaol has shown effective anticancer activity in different animal tumor models, but the dosage, route of administration, and mechanism of action are different when treating different types of tumors, as shown in Table 2.

**Table 2:** 6-Shogaol in vivo anticancer study (“↑” represents increase/ promotion/activation while “↓” represents inhibition /reduction/inactivation.)

Cancer models	Cancer cell lines	Tumor xenograft sites	Dose/duration and route of administration	Mechanisms	Tumor suppression efficacy	Ref.
Oral cancer	DMBA Induces Canceration	Male golden Syrian hamsters aged 8–10 weeks	20 mg/kg/day thrice A week for 16 weeks Not specified	Inhibit transcriptional Activate NF-KB/AP-1	Inhibition of cell Proliferation and inflammation	[56]
Non-small cell lung cancer	NCI-H1650	Female BALB/c (nu/nu) mice	10 or 40 mg/kg/day Three times a week for 3 weeks i.p	Ki-67↓ cyclin D1↓ p-AKT↓ STAT3↓	Inhibit NCI-H1650 lung cancer cell growth	[49]
	A549	Nu/J nude mice on both sides	10 or 30 mg/kg/day Five times a week i.p	Induce apoptosis: GSH↑ p53↑ PUMA↑ Bcl-2↓ Bcl-XL↓	Inhibit cell proliferation	[47]
Prostate cancer	PC-3	the right flanks of athymic nude mice	250 mg/kg 28 days oral gavage	Anti-tumor proliferation	Inhibition of tumor proliferation	[57]
	HMVP2	the flank of Isogenic FVB/N male mice	50 and 100 mg/kg every other day for 32 days i.p	pSTAT3^Y705^↓ cyclin D1↓ survivin↓	The tumor weight was reduced by 48% and 65% at both doses, respectively	[58]
Colon cancer	HCT-116 SW-480	Female athymic nude mice	15 mg/kg/day The day after Cancer cell Injection and every other day 28 days i.p	Induce apoptosis:p53↑ bcl-2↓ BAD↑ Caspase3↑ Caspase8↑ Caspase9↑ Induce cell cycle arrest in the G2/M phase:p53↑ p21↑cdc2↓ cdc25A↓	6-Shogaol significantly inhibited the proliferation of HCT-116 cells	[59]
Cervical cancer	HeLa	The right axilla of Female BALB/c 18 nude mice	0,12.5,and,50 mg/kg/day One time every day 21 Days Oral gavage	Induce apoptosis: p-PI3K↓,p-Akt↓ p-mTOR↓ Induce G2/M phase arrest Inhibition of metastasis	Inhibition of tumor growth in a dose dependent manner	[13]
Leukaemia	CCRF-CEM	Subcutaneous Of female athymic nude mice	80 mg/kg/day five times a week 60 days i.p	Induce apoptosis: p53↑ ROS↑ FASN↓	Inhibition of tumour growth	[60]
Liver cancer	SMMC-7721	Inoculated subcutaneously into SCID mice	10 mg/kg or 50 mg/kg/day 28 days i.p	Induce apoptosis: ER stress↑ p-eIF2α↓ CHOP↑ caspase↑ PARP↓	The tumor volume of mice in the 6-shogaol administration group was significantly smaller than that of carrier control mice at two treatment doses (10 mg/kg and 50 mg/kg)	[38]
	HepG2	Subcutaneously inoculated into the right rib of BALB/c nude mice	50 mg·kg/day once every 2 days 35 days Intraperitoneal injection	Induce apoptosis AKT/mTOR↓ MRP1↓ Induce G0/G1 phase c arrest: AKT/mTOR/MRP1↓ CyclinA2↓ Cyclin D1↓ Cyclin E1↓	Tumor volume was lower in the 6-shogaol group than in the control group	[61]
Pancreatic cancer	PANC-1	the right flank of pathogen-free male BALB/c immunodeficient nude mice	50 mg/kg once daily 28 days i.p	Inhibit NF-KB signaling	Effectively inhibits the growth of pancreatic cancer cells	[52]

## The therapeutic mechanism of 6-shogaol on cancer

**Fig. 2:**
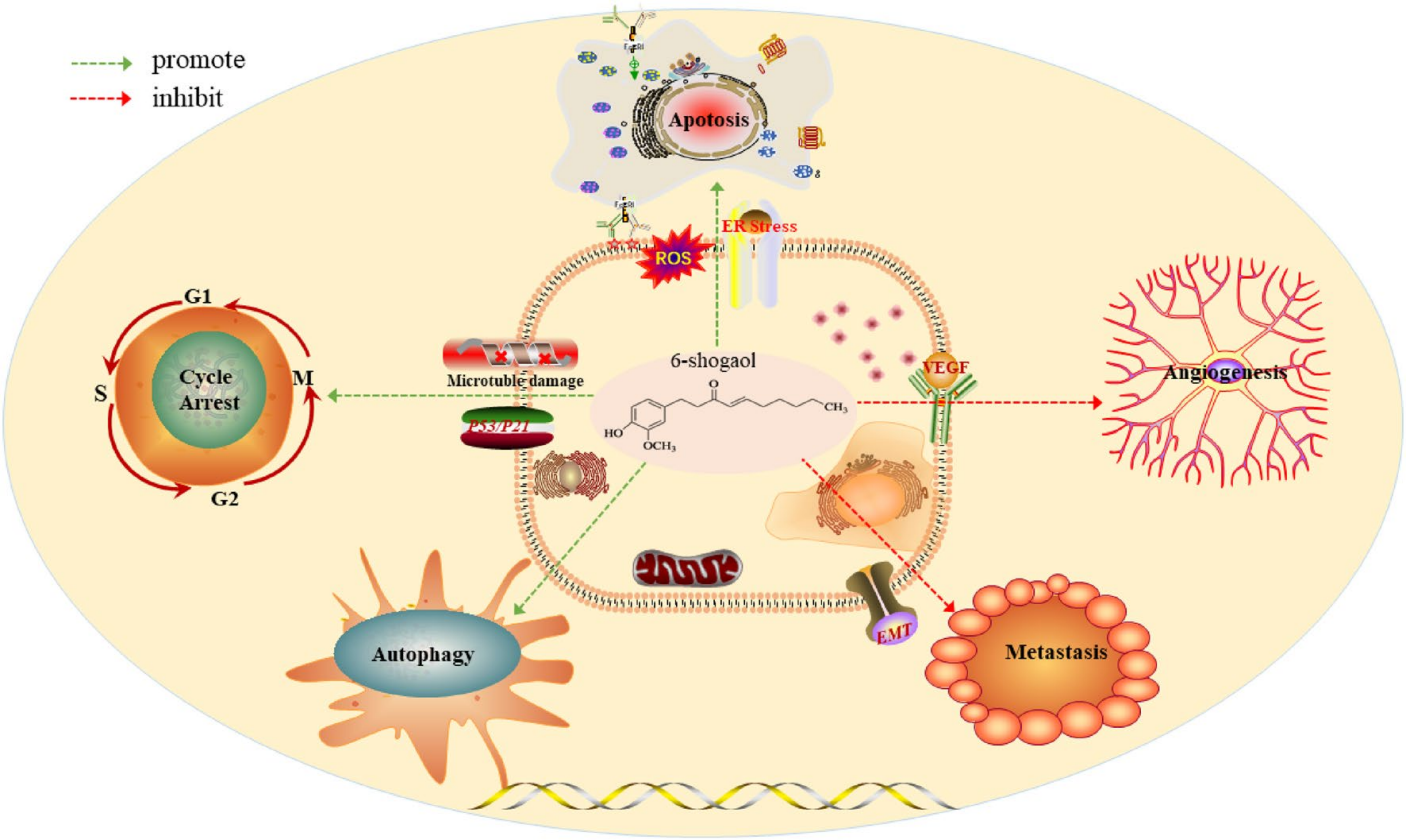
6-Shogaol anti-tumor mechanism

The anti-tumor mechanism of 6-shogaol is related to many biochemical processes, including apoptosis, autophagy, cell cycle arrest, EMT/migration/invasion/metastasis, and angiogenesis (Fig. 2).

### Induce cancer cell apoptosis

Apoptosis, autophagy and necrosis are the three main pathways of cell death, with apoptosis considered to be the most important cause of death in all cell lineages [62]. Chromatin condensation, membrane blistering, DNA breakage and cleavage of DNA repair protein PARP are the main features of apoptosis [62]. Cell apoptosis is a highly regulated process of cell death, generally mediated by endogenous and exogenous factors [63]. The main reason why cancer cells continue to proliferate is believed to be their ability to escape programmed cell death. Therefore, inducing apoptosis has become a major strategy for cancer treatment [64].

**Fig. 3:**
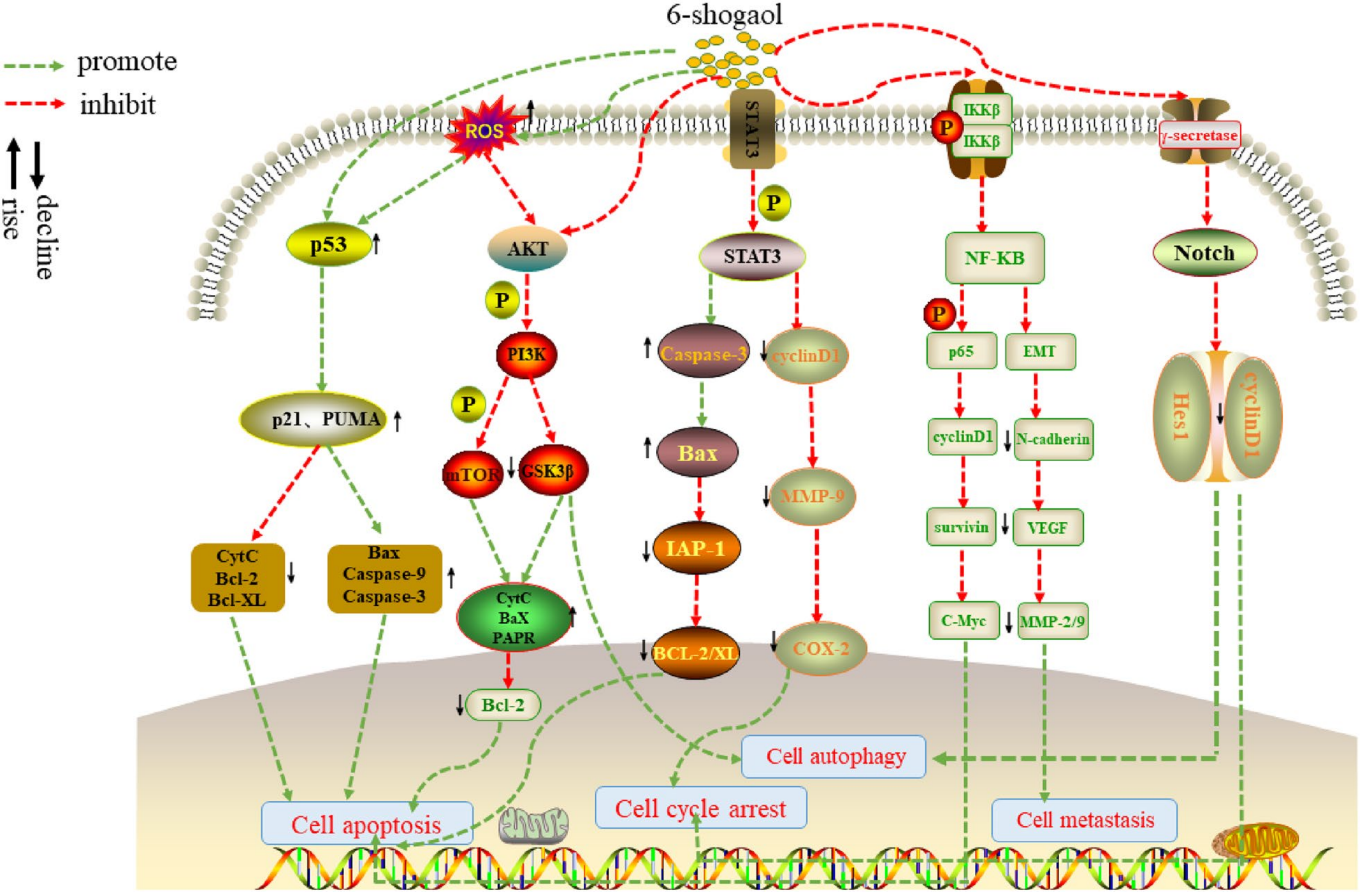
Signaling pathways involved in the anticancer effects of 6-shogaol

**Fig. 4:**
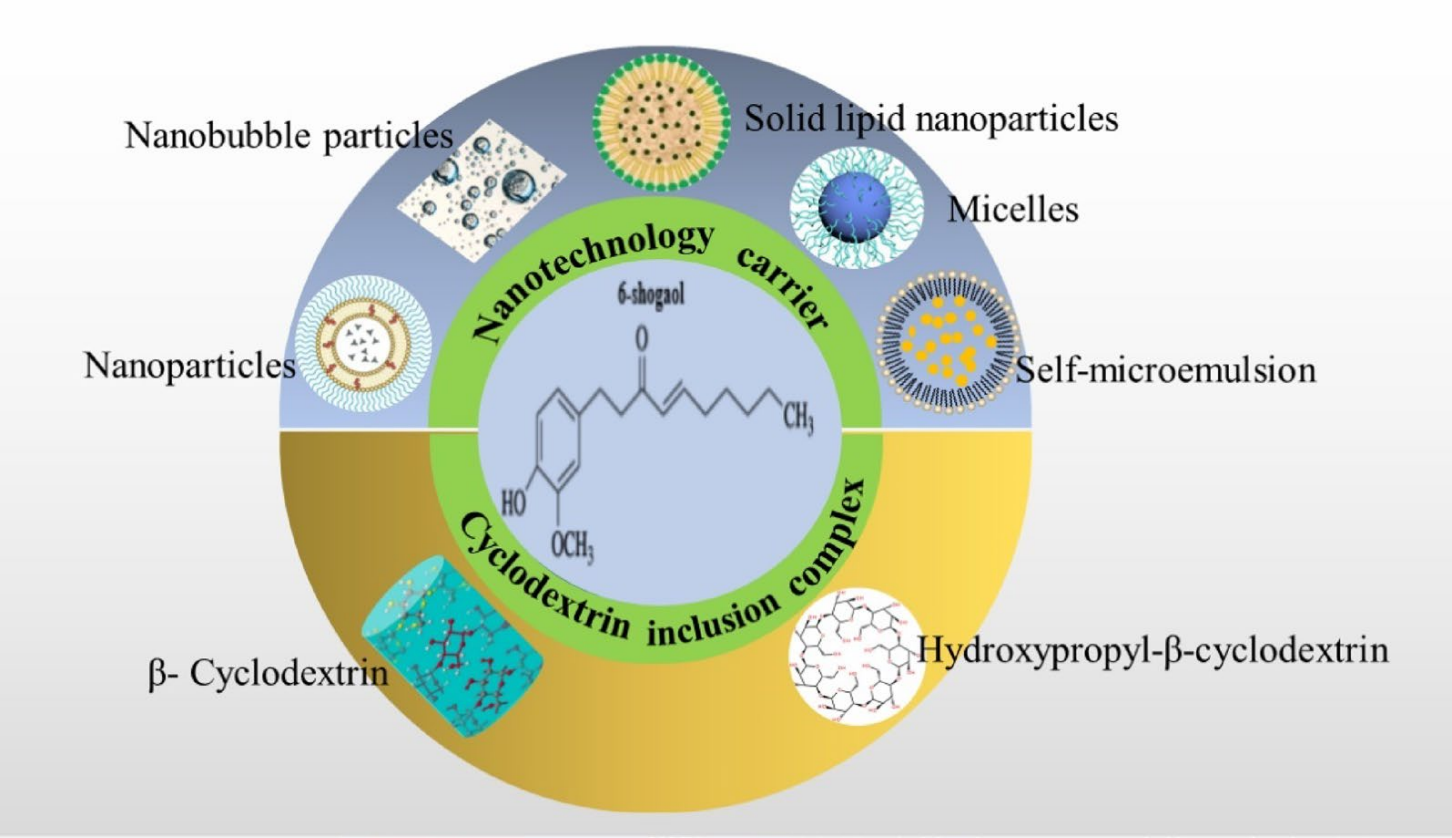
Potential delivery systems for 6-shogaol

Through the comprehensive summary of the anticancer research on 6-shogaol, it is found that 6-shogaol is a potent inducer of apoptosis in many cancer cells. 6-shogaol promotes apoptosis by regulating related signal pathways and protein expression. Acute lymphoid leukemia Nalm-6 cells induced massive apoptosis after 6-shogaol intervention and upregulation of p53 was found to mediate the onset of apoptosis [60]. In the oral squamous cell carcinoma model, 6-shogaol induced cancer cell apoptosis by inhibiting the PI3K/AKT/mTOR signaling pathway [44]. In a hepatocellular carcinoma model, 6-shogaol induced apoptosis by inhibiting the AKT/mTOR/MRP1 pathway, thus exhibiting apoptotic phenomena such as smaller nuclei, condensation of chromatin around the nuclear membrane, and rupture of the nucleus [15, 61]. In pancreatic cancer cells, 6-shogaol has been confirmed to induce apoptosis by inhibiting NF kB and its downstream target genes [52]. This discovery was also confirmed in the research of Tan et al. In addition, they also found that 6-shogaol can induce PPAR γ Activation to inhibit NF kB signal mediated apoptosis [43]. In addition, inhibition of STAT3 activation by 6-shogaol is also the main way to promote apoptosis [58, 65, 66]. In addition to directly or indirectly inhibiting the phosphorylation level of STAT3 protein, 6-shogaol can also inhibit its translation ability [29]. In addition, it has also been found that 6-shogaol promotes apoptosis in cancer cells by mediating endoplasmic reticulum (ER) stress and reactive oxygen species (ROS) overexpression. As these two pathways seem to be the main pathways of 6-shogaol to promote apoptosis, they will be described in detail below (Figs. 3, 4).

#### Induce ER stress to promote apoptosis

ER stress is the result of endoplasmic reticulum dysfunction leading to massive accumulation of unfolded proteins. Dysfunction of the endoplasmic reticulum in maintaining cellular homeostasis has been reported to be associated with a hypoxic and hypotrophic tumor microenvironment. ER stress is a signaling pathway known as the “unfolded protein response” (UPR), which is regulated by three sensors: PKR-like endoplasmic reticulum-associated kinase (PERK), activating transcription factor 6 (ATF6), and inositol-requiring enzyme-1 (IRE1) [38]. It is worth noting that sustained ER stress mediates apoptosis through ATF6, PERK, and IRE1. Therefore, targeting the promotion of ER stress-induced apoptosis in cancer cells has become a possible anticancer strategy [64]. In hepatocellular carcinoma, 6-shogaol triggers endoplasmic reticulum stress response through inhibition of PERK/eIf2α signaling pathway thereby inducing apoptosis in SMMC-7721 cells. According to experimental studies, 6-shogaol was found to induce apoptosis by regulating the unfolded protein response (UPR) sensor PERK and its downstream target eIF2α, while being insensitive to the other two sensors ATF6 and IRE1 [38]. In a hepatocellular carcinoma model, cancer cells exposed to 6-shogaol showed a time-dependent increase in ER stress-related proteins, including GRP-78/Bip and GRP-94, and the expression of phospho-PERK and phospho-eIF2α showed an increase followed by a decrease, suggesting that 6-shogaol mediates the onset of ER stress. However, it was found that upregulation of these ER-related kinases did not lead to significant apoptosis of hepatocellular carcinoma cells, but rather prolonged 6-shogaol treatment-induced inhibition of eIF2α phosphorylation was related to an increase in apoptosis. eLF2α phosphorylation underwent a role shift from a cytoprotective to a pro-apoptotic state with continued 6-shogaol treatment. In addition, 6-shogaol significantly upregulated the expression of CHOP, which is an effector of apoptosis and directly mediates apoptosis [38]. Consistent findings were also obtained by Wu et al. in their study of the same tumor model. Furthermore, they proposed that 6-shogaol-induced ER stress is involved in autophagy and that the induced autophagy exerts toxic effects on cancer cells [15]. In human lipoma SW872 cells, 6-shogaol induced not only the upregulation of GRP-78, eIF-2α, and CHOP but also increased the expression of ATF-4 [29]. In addition, it has been reported in the literature that 6-shogaol binds to the chymotrypsin-like subunit and inhibits proteasome activity, thereby inducing endoplasmic reticulum stress through the accumulation of polyubiquitinated proteins to promote apoptosis in cancer cells [37].

#### Induce ROS overexpression to promote apoptosis

According to the literature survey, ROS, highly expressed in tumor cells, can destroy deoxyribonucleic acid (DNA), proteins and lipids, eventually leading to apoptosis [67, 68]. Based on this direction, finding an effective method that can increase the concentration of ROS in cancer cells without affecting normal cells is a promising anticancer method.

Encouragingly, several studies have found that 6-shogaol appears to have this therapeutic potential. In a fibrosarcoma cell (HT1080) model, the use of 6-shogaol at concentrations of 30–70 uM was found to significantly inhibit cancer cell viability, while an increase in ROS in HT1080 cells was observed in a dose-dependent manner, with a more pronounced increase at a concentration of 50 μM [69]. However, what was most pleasing was that noncancerous fibroblasts did not show this change after the 6-shogaol treatment. In addition, when the cancer cells were treated with 5 mM NAC (N-acetylcysteine) in combination with 6-shogaol, NAC partially reversed this change and also reduced the virulence to the cancer cells. This suggests that 6-shogaol-triggered cancer cell death is associated with an increase in ROS [69]. This encouraging finding was echoed in a study of 6-shogaol in acute leukemia. The group examined ROS levels in Nalm-6 cells when treated with 6-shogaol and found that ROS expression was much higher in 6-shogaol-treated cells than in other controls, and found that the addition of the ROS scavenger NAC reversed the therapeutic effect of 6-shogaol. In addition, through further studies, 6-shogaol causes DNA damage by mediating the overexpression of ROS in leukemic cells, leading to the activation of p53 signaling pathway to elevate the expression of proapoptotic genes and genes participated in cell cycle arrest, such as PUMA and p21. In addition, high expression of ROS in cancer cells may also induce apoptosis by inhibiting fatty acid synthase (FASN) [70]. As a pro-oxidant, 6-shogaol increases the production of ROS in human laryngeal carcinoma (Hep-2) cells, leading to a loss of mitochondrial membrane protein potential and thus a decrease in the Bcl-2/Bax ratio. Increased pro-apoptotic factor Bax mediates the release of cytochrome-c from mitochondria, which leads to caspase-9 and caspase-3 activation to induce apoptosis in Hep-2 cells [71]. In addition, a novel mechanism of 6-shogaol-mediated ROS-triggered apoptosis was identified in a kidney cancer cell model (Caki), 6-shogaol promoted TRAIL-induced apoptosis more sensitively by increasing ROS production, mediating cytochrome C release and decreasing c-FLIP(L) expression [45]. In a recent report on cervical cancer cells, 6-shogaol-mediated high expression of ROS may inhibit the PI3K/Akt/mTOR signaling pathway, thus showing anti-tumor growth activity [13]. Yi et al. proposed that ROS are autophagy inducers and that 6-shogaol induces autophagy in cancer cells by causing high expression of ROS in cancer cells [72].

### Regulates growth cycle arrest

The abnormality of the cell cycle regulation mechanism is a Common characteristic of all types of cancer. The claim that cancer is a cell cycle disease is increasingly recognized, as cells continue to divide in a disorderly manner, contributing to the rapid growth and progression of tumors [73]. A complete cell cycle consists of four phases: pre-DNA synthesis (G1), DNA synthesis phase (S), late DNA synthesis (G2), and mitosis (M). Aberrant activation of cyclins as a driving force for tumorigenesis [73]. Therefore, targeted regulation of cell cycle arrest is a potential anticancer target [74]. In recent years, numerous experimental studies have shown that 6-shogaol can induce G2/M phase arrest of tumor cells by regulating different signaling pathways, triggering cell death.

In non small cell lung cancer (NSCLC), 6-shogaol dose dependently blocks the progression of G2/M phase in squamous NCI-H226 cells [75]. In colon cancer HCT116 and neuroblastoma SH-SY5Y cells, 6-shogaol causes G2/M phase arrest through attenuation of cyclin and spindle assembly checkpoint proteins, including mdc20, mad2, and survivin. Checkpoint proteins serve as the central regulator of cell cycle progression, monitoring all defects in cells from one cell cycle to the next. Some studies have found that after 24 h of 6-shogaol treatment of HCT116 cells, significant downregulation of checkpoint proteins in the cell cycle was observed, including phosphorylated forms of e-cdk1, e-cyclinB1, and e-cdc25C, as well as cdk1 and cdc25C, where cdk1/cyclinB is the key checkpoint for G2 phase cell populations to enter the M phase. This suggests that 6-shogaol causes C2/M phase arrest in cancer cells by reducing checkpoint protein expression. In addition, 6-shogaol-induced mitotic abnormalities may also be closely related to the direct targeting of 6-shogaol to tubulin [76]. Bawadood et al. also suggested that 6-shogaol may induce G2/M-arrest by interfering with cellular microtubule structures [35]. Microtubules are an integral component of the cell and participate in the formation of the cytoskeleton, thereby performing a variety of cellular functions, including mitosis. 6-shogaol interacts with the SH group of the cysteine residue in microtubule protein, thereby binding to microtubule protein, causing microtubule damage, leading to blocking of the G2/M phase of cancer cells [77]. In another study on colon cancer HCT-116, 6-shogaol was found to cause growth arrest in G2/M phase by mediating the upregulation of p53, p21 and GADD45α and the downregulation of cdc2 and cdc25a. Notably, the p53/p21 pathway is thought to be the main pathway of 6-shogaol-induced G2/M cell cycle arrest [59].

### Induce cancer cell autophagy

Autophagy is lysosome-mediated cellular self-digestion, which is an evolutionarily conserved normal physiological process of self-degradation and also a cell quality control mechanism [78]. Autophagy is like a double-edged sword in cancer, which plays a dual role of promotion or inhibition in different types of tumors and different stages and backgrounds of tumor development. Autophagy has become an effective target for cancer therapy because of its potential to regulate cell death [79]. According to literature studies, 6-shogaol triggers cell death by targeting autophagy (both autophagy induction and autophagy inhibition), but mainly by inducing autophagy.

In a breast cancer model, 6-shogaol significantly reduced the expression of autophagy-related genes beclin-I and LC3-II in breast cancer cells, and the formation of autophagosomes in tumour cells was inhibited thereby inducing cell death. This suggests that 6-shogaol may force these cells to die by inhibiting autophagy [35]. However, in the same breast cancer model, Ray Anas et al. reached the opposite conclusion to the above. This study found that 6-shogaol triggered the death of breast cancer cells by inducing autophagy and that autophagy was a predominant mode of 6-shogaol-mediated cell death. Treatment of breast cancer MCF-7 cells with 6-shogaol for 48 h induced the generation of large numbers of cytoplasmic vesicles and induced lipid modification from LC3-I to LC3-II, indicating that autophagy occurred. In addition, treatment of MCF-7 cells with 6-shogaol (1–9 uM) in combination with the autophagy inhibitor chloroquine (5 uM) significantly reduced the percentage of cancer cell death compared to 6-shogaol alone, further confirming that 6-shogaol-induced autophagy is one of the major mechanisms leading to tumour cell death [12]. In a human non-small cell lung cancer model, 6-shogaol induces autophagy in A549 cells through inhibition of the Akt/mTOR pathway resulting in anti-proliferation. During the initial stage of autophagy, mTOR expression is inhibited, leading to the formation of bimembrane vesicles. The membrane encapsulates some organelles or tumor proteins, forming autophagosomes, which then fuse with lysosomes to form autophagosomes, thereby degrading these encapsulated organelles and tumor proteins. According to the study, 6-shogaol inhibition of the AKT signalling pathway was associated with a reduction in phosphorylation of downstream targets GSK-3β and FKHR [46]. In pancreatic cancer, liver cancer, cervical cancer, and colorectal adenocarcinoma, 6-shogaol also showed anti-tumor proliferation activity by inducing autophagy. In the study of 6-shogaol anti proliferation of pancreatic cancer, it was found that 6-shogaol increased the LC3-II/LC3-I ratio of human pancreatic cancer Panc-1 cells in a time-dependent and dose-dependent manner, which indicated that the anti proliferation effect of 6-shogaol was related to the induction of autophagy. In this tumor model, 6-shogaol activates AMPK, a positive regulator of autophagy, while inhibiting mTOR, a negative regulator of autophagy. When cells treated with 6-shogaol were treated with the autophagy inhibitors 3-methyladenine and chloroquine, tumour cell death was significantly reduced. Thus, this is further evidence that 6-shogaol-induced pancreatic cancer cell death appears to be induced by autophagic cell death [72]. Autophagy induced by 6-shogaol also plays a cytotoxic role in hepatoma cells. After treatment of HepG2 cells with 6-shogaol, a significant increase in the expression of LC3-II was observed, and a causal relationship was found between the high expression of LC3-II and the reduced impact on cell viability. In addition, 6-shogaol-induced autophagy was also highlighted to be associated with ROS production and ER stress in this cancer model [15]. 6-shogaol-induced autophagy as a major cell death type was confirmed in the human colorectal cancer cell line HT-29 [42]. In addition, it has been suggested that 6-shogaol induces autophagy and cycle arrest in cancer cells, both of which promote each other and thus jointly mediate cancer cell death.

### Inhibit cancer cells metastasis

Tumour metastasis is a major cause of death in cancer patients, and EMT marker proteins are an important factor in enhancing cancer cell invasion and metastasis. Matrix metalloproteinases (MMP) are key enzymes in extracellular matrix (ECM) and basement membrane catabolism and show high expression in tumor cells, which help tumor metastasis by degrading extracellular matrix (ECM) and basement membrane. Epithelial-mesenchymal transition (EMT) is a process by which epithelial cells acquire an extreme ability to migrate and invade, a process that is closely related to tumor metastasis, invasion, and chemoresistance [80]. In oral cancer OSCC cell lines, 6-shogaol at a concentration of 8 μM inhibited the migration and invasion of 60% of cancer cells, which was found to be related to the downregulation of EMT related proteins by 6-shogaol, including E-cadherin and N-cadherin [44]. In breast cancer models, 6-shogaol was shown to attenuate the expression of the EMT marker gene Vimentin. In addition, it was found that 6-shogaol also inhibited the invasive migration of MDA-MB-231 cells by down-regulating the expression of Gli1, a factor downstream of Hh signalling, which is strongly associated with the development, progression and metastasis of breast carcinomatosis. Furthermore, the study also found that the Gli1 gene may be related to the progression of EMT [81]. In gastric cancer, 6-shogaol intervention in gastric cancer BGC-823 cells significantly affects the expression of EMT related proteins and regulates E-cadherin protein, MMP-2, and MMP-9. In addition, 6-shogaol inhibits cancer cell metastasis by inhibiting the activation of NF kB signal to down-regulate MMP-9, MMP-7, and MMP-13 [36].

### Inhibit tumor angiogenesis

The process of developing new blood vessels from existing ones by germination is known as angiogenesis and has been shown to promote the growth and proliferation of solid tumours [82]. 6-shogaol was found to inhibit the secretion of the angiogenic inducers vascular endothelial growth factor (VEGF) and interleukin-8 (IL-8) by blocking the activation of NF-kB in ovarian cancer cells, thereby inhibiting angiogenesis [83]. 6-shogaol treatment of aortic rings in a mouse model of hepatocellular carcinoma not only strongly inhibited the number of VEGF-triggered sprouts but also interfered with pre-existing angiogenic sprouts [40]. Bischoff-Kont et al. attributed the inhibitory effect of 6-shogaol on VEGF-induced angiogenic germination in part to the downregulation of VEGFR2 levels [84]. Weng et al. showed that 6-shogaol inhibited the expression of MMP-2/-9 and urokinase-type fibrinogen activator (uPA) in Hep3B cells by suppressing NF-kB, MAPK, PI3k/Akt, and STAT3 activity, thereby blocking vascular regeneration [40]. In addition, studies have shown that 6-shogaol can effectively inhibit tumor angiogenesis, which is related to its own structure, Michael receptors, and the number of Michael receptors is correlated with their antiangiogenic activity [85].These studies suggest that 6-shogaol is a potent inhibitor of tumor angiogenesis.

## The regulation of 6-shogaol on cancer related signal pathways

### Activate p53 signaling pathway

P53 is a key tumor suppressor that plays an irreplaceable role in cancer prevention and development [86], Stimuli such as DNA damage, hypoxia and expression of certain oncoproteins (e.g. Myc, Ras) activate the p53 signaling pathway, which in turn activates pro-apoptotic factors (e.g. Bax) in its downstream targets while inhibiting anti-apoptotic factors (e.g. Bcl-2) to induce apoptosis in cancer cells.

6-shogaol is an effective inducer of the p53 signaling pathway, activating p53 to mediate cancer cell apoptosis. Upregulation of the p53 protein is involved in the expression of apoptosis-related genes, including p21, PUMA, Bax, Bcl-xl and Bcl-2. PUMA is a transcriptional target of p53 and PUMA assists in promoting apoptosis by binding to Bcl-xl to disrupt its binding to p53 [60]. P21 is not only a downstream target of p53, but also binds directly to p53 to form the p53/P21 complex. This complex binds to Bcl-2 family proteins to promote the expression of Bax [87]. Furthermore, in addition to the direct activation of p53 signalling, an indirect activation pathway exists for 6-shogaol. 6-shogaol triggers activation of p53 signaling by increasing the concentration of ROS in cancer cells [60]. In addition, 6-shogaol activates p53 signaling by depleting GSH in cancer cells. Subsequently, the expression of Bcl-XL and Bcl-2 was suppressed and cytochrome C, Caspase-3 and Caspase-9 were upregulated, triggering apoptosis [47]. This finding was also confirmed in a study of 6-shogaol on oral squamous cell carcinoma cell lines (OSCC). p53, Bax and cleaved caspase-3 expression were significantly increased in 6-shogaol-treated OSCC cells, while Bcl-2 expression was attenuated, ultimately causing apoptosis in OSCC cells [44]. Furthermore, Uddin et al. found that 6-shogaol promoted apoptosis by increasing TRAIL-mediated p53 protein expression in a combination regimen of 6-shogaol and TRAIL [88]. Based on these experimental studies it was shown that the p53 signaling pathway is an important pathway for 6-shogaol to induce apoptosis in cancer cells.

### Inhibit AKT signaling pathway

Akt is a member of the serine/threonine protein kinase family, also known as protein kinase B (PKB) [89]. It is often activated in various types of cancer and thus promotes further tumor development and drug resistance [89]. There are three main isoforms of Akt (Akt1, Akt2 and Akt3). These three isoforms have high structural similarity, all having amino-terminal homology (PH) structural domains, kinase structural domains, and carboxy-terminal structural domains (HM) as regulatory structural domains [90]. Akt, as the central point of the PI3K/Akt/mTOR pathway, regulates multiple cellular processes, including apoptosis. The PI3K/Akt/mTOR signaling pathway exhibits excessive activation in different types of cancer, which has been fully confirmed. Once this signaling pathway is activated, it leads to the upregulation of various oncogenes. 6-shogaol has been found to inhibit oncogene upregulation and trigger apoptosis by inhibiting AKT signaling.

In non-small cell lung cancer, 6-shogaol inhibits AKT kinase activity by binding to the variable configuration site of Akt, thereby inducing cleavage of the apoptosis markers Caspase-3 and -7 [49]. In oral squamous cell carcinoma, 6-shogaol binds directly to Akt as a ligand, thereby blocking this signaling. Subsequently, the expression of p-PI3K, p-AKT, p-mTOR and GSK3β, a molecule downstream of Akt, was downregulated in a dose-dependent manner, inducing massive apoptosis [44]. In cervical, 6-shogaol triggers the mitochondrial apoptotic pathway by inhibiting the PI3K/Akt/mTOR signaling. It is well known that the decrease of mitochondrial membrane potential is a sign of early apoptosis. The treatment of Hela and SiHa cells with 6-shogaol resulted in the loss of mitochondrial membrane potential of these two cells, and increased the expression of cytochrome c, PARP and Bax, while reducing Bcl-2. In addition, 6-shogaol has synergistic effect with PI3K inhibitor (LY294002) to promote apoptosis of tumor cells, while it has antagonism with PI3K agonist (IGF-1) to eliminate the induction of apoptosis, which further indicates that 6-shogaol is a potential inhibitor of PI3K/Akt/mTOR signaling pathway [13].

### Inhibit STAT3 signaling pathway

Signal transducer and activator of transcription3 (STAT3) is a member of the STAT protein family and plays the dual role of signal transducer and transcription factor [91]. In many solid tumors, STAT3 is activated in groups, providing proliferation signals to cancer cells. According to reports, the activation of STAT3 signaling is the main intrinsic pathway for the occurrence and development of various cancers [92]. Genetic silencing or artificial inhibition of STAT-3 induces apoptosis [93]. According to the literature survey, STAT3 signaling is also one of the pathways targeted by 6-shogaol and 6-shogaol can inhibit STAT3 activation to achieve an anti-proliferative effect [94].

In the liposarcoma cell model, 6-shogaol induces apoptosis by inhibiting STAT3 phosphorylation in SW872 liposarcoma cells, leading to caspase-3 activation and PARP cleavage [29]. In breast and prostate cancer cells, 6-shogaol downregulates STAT3-regulated gene expression by inhibiting the group activation of STAT3, including Bcl- xl, Bcl-2, IAP-1, survivin, cyclin D1, MMP-9 and COX-2, thereby inhibiting cell proliferation, leading to G1-G0 phase arrest and inducing cell apoptosis. In addition, further studies revealed that STAT3 activation was regulated by upstream kinases such as Jak2 and Src. 6-Shogaol strongly inhibited the native phosphorylation of STAT3 by suppressing Jak2 and c-Src activity and nuclear translocation of STAT3 on tumor cells, thereby inhibiting STAT3 signaling [65]. Studies have found that the activation of STAT3 is also closely related to the secretion of CC-Chemical Factor Ligand 2 (CCL2). CCL2 generally exhibits high expression levels in cancer cells, thereby increasing the migration and invasion of cancer cells. The increased phosphorylation of STAT3 will enhance the expression of CCL2 in human lung cancer A549 and breast cancer MDA-MB-231 cells. When 6-shogaol was used to treat A549 and MDA-MB-231 cells, this effect was completely reversed, indicating that 6-shogaol downregulated the expression of CCL2 by inhibiting STAT3 signaling [66].

### Inhibit the NF-KB signaling pathway

Nuclear factor Kappa-B (NF kB) is a key regulatory factor for cell survival, consisting of five members: NF kB1 (p50 and its precursor p105), NF kB2 (p52 and its precursor p100), Rel (c-Rel), RelA (p65), and RelB [95]. NF-kB signaling molecules are well known for their anti-apoptotic effects and have attracted much attention and research. NF-kB signaling drives carcinogenesis in solid and hematologic malignancies, disease recurrence, and treatment resistance [96]. Its importance in the progression of various cancers makes it an excellent target for cancer therapy [97]. NF kB is also considered as one of the targets of many natural active products, such as curcumin, resveratrol, epigallocatechin, gallic acid, carotenoids and 6-shogaol [36].

Among them, several literatures reported the mechanism of 6-shogaol inhibiting tumor growth by targeting NF-kB signaling molecules. In 7,12-dimethylbenz[a]anthracene (DMBA)-induced oral carcinogenesis (HBP) in hamsters, DMBA-induced hamsters exhibited abnormally high expression of NF-KB-p65, IKKβ, c-Jun and c-Fos, which triggered signals for cancer cell proliferation. NF-kB-p65 activation translocates from the cytoplasm to the nucleus and participates in the activation of hundreds of target genes, thereby promoting tumour development. 6-shogaol impairs the activation of NF-kB signaling by inhibiting the phosphorylation/or degradation of IKKβ and IKB-α, thereby blocking the nuclear translocation and phosphorylation of NF-kBp65 [56]. In addition, 6-shogaol also inhibits tumour cell proliferation and metastasis by inhibiting IkB-α phosphorylation and proteasomal degradation, delaying nuclear translocation of p65 and inhibiting NF-kB transcriptional activity [43]. The transcriptional activation of NF-kB has been confirmed to be associated with the high expression of inflammatory markers COX-2 and iNOS, and inflammation is considered one of the characteristics of tumor development. 6-Shogaol inhibits tumor progression by down-regulating the expression of COX-2 and iNOS induced by DMBA in hamsters by inhibiting NF-kB activation [56]. In addition, IkB kinase inhibitor (IKK) is a major upstream regulator of NF-kB, which is susceptible to activation by pro-inflammatory cytokines, including TNF-α and IL-1. Once IKK is activated and phosphorylated, it causes phosphorylation and proteasomal degradation of IKBα, which activates NF-kB signaling. In human prostate cancer cells, 6-Shogaol (40 μmol/L) treatment of prostate cancer PC-3, DU145 and LNCaP cells reduced the levels of TNF-α-induced p-NF-kBp65^ser536^ and the negative regulator p-IKBα^S32/36^. In addition, the 6-shogaol treatment also down-regulated NF-kBp65 expression in the nuclei of LNCaP and DU145 cells. The inhibition of NF-kB by 6-shogaol was also accompanied by down-regulation of its downstream target protein genes cyclinD1, survivin and cMyc [58]. Inhibition of 6-shogaol targeting of NF-kB signalling molecules is a key step in the treatment of several diseases caused by inflammation, such as acute kidney injury [98], CCL4-induced liver fibrosis [99] and cardioprotection [100].

### Inhibit the notch signaling pathway

The notch signaling pathway regulates cell survival, proliferation and differentiation and is in a disorder state in many types of cancer. Hes (Hairy Enhance of Split) and Hey (Hairy/Enhancer of Spit YRPW motif) are the two main effectors of transcriptional regulation by this signaling pathway [101]. 6-Shogaol exerts anti-cancer effects by inhibiting the aberrant activation of Notch signaling. In MCF-7 cells, 6-Shogaol reduced the expression of the target genes D1 and Hes1 by blocking Notch signaling, ultimately reducing the proliferation and survival of breast carcinoma cells [35]. In the same breast cancer model, the combination of 6-Shogaol with the γ-secretase inhibitor DAPT caused a more pronounced reduction in Notch and its target proteins compared to DAPT or 6-shogaol alone. Thus, Anasuya et al. suggested that 6-shogaol may mediate the inhibition of Notch signaling through inhibition of γ-secretase [102].

## Synergies

Although many kinds of anticancer drugs have been widely used in clinical practice, their side effects and drug resistance limit the effectiveness and clinical application of these drugs, which is an obstacle on the way to the successful treatment of cancer. More and more studies have shown that the combination of clinical chemotherapy drugs and 6-shogaol can reduce or eliminate the side effects of some chemotherapy drugs and improve the drug resistance of commonly used chemotherapy drugs by targeting the molecular mechanism of regulating tumor cells. 6-Shogaol has been shown to produce good synergistic effects in combination with the following chemotherapeutic agents.

### Cisplatin

Cisplatin is one of the earliest and most effective chemotherapy drugs. It is widely used in the treatment of various solid tumors such as cervical, lung [103], stomach [104], ovarian cancer [105] and other types of cancer [103, 106]. Cisplatin is highly effective therapeutically, but high doses induce serious complications such as nephrotoxicity, ototoxicity and neurotoxicity. These toxic effects limit the effectiveness and clinical use of cisplatin [107]. As is well known, cancer patients treated with cisplatin can induce systemic and renal inflammation, leading to severe kidney damage. Studies have confirmed that 6-shogaol exhibits good protective effects on cisplatin induced acute kidney injury (AKI). 6-Shogaol inhibits two major types of renal tubular cell death in cisplatin-induced renal injury by downregulating the expression levels of TNF-α and IL-6 in serum and kidney and decreasing the concentrations of cisplatin-induced chemokines, MCP-1 and CCL5 [108]. This suggests that the combination of cisplatin and 6-shogaol may reduce or eliminate the toxic effects of cisplatin, resulting in an efficient and less toxic treatment.

### Gemcitabine

The combination of 6-shogaol and gemcitabine shows great potential in the treatment of pancreatic cancer. Gemcitabine is a commonly used drug approved by FDA for clinical treatment of pancreatic cancer, but its toxicity and drug resistance lead to poor therapeutic effect. According to the results of the combination of gemcitabine and 6-shogaol, 6-shogaol not only increased the sensitivity of pancreatic cancer cells PANC-1 and BxPC-3 to gemcitabine, but also enhanced the anti-cancer effect of gemcitabine by inhibiting TLR4/NF-kB signaling. The interaction of gemcitabine with cancer cells induces the DNA binding ability of NF kB, thus mediating the activation of NF kB signals. It is worth noting that 6-shogaol can inhibit gemcitabine induced NF kB activation, and down regulate the expression of proteins that contribute to chemotherapy resistance of pancreatic cancer, including cyclin D1, COX-2, survivin, Bcl-2, XIAP, cIAP-1 and MMP-9, ultimately leading to growth arrest and apoptosis [52].

### Imatinib (IMA)

Chronic myeloid leukaemia (CML) is a clonal haematopoietic malignancy in which the formation of BCR-ABL fusion proteins is the most distinctive feature. Imatinib (IMA) is a BCR-ABL tyrosine kinase inhibitor (TKI) that is highly sensitive in patients with newly diagnosed CML. However, as the duration of treatment increases, the emergence of IMA resistance becomes a major challenge in the treatment of CML [109]. IMA resistance has been reported to involve different mechanisms, including apoptosis, autophagy and drug efflux transport proteins such as MDR-1 and MRP-1 [110]. Excitingly, in a study by Ozkan et al. it was found that 6-shogaol overcame the resistance of cancer cells to IMA and synergized with IMA to promote apoptosis. MDR-1 and MRP-1 were expressed at high levels in K562R (imatinib-resistant) and K562S (imatinib-sensitive) cells. 6-shogaol treatment did not reduce the expression of MDR-1 in either cell type, but the expression level of MRP-1 mRNA, a multidrug resistance-associated protein, was significantly reduced in K562S cells. In addition, 6-shogaol induced apoptosis in K562S and K562R cells by triggering the mitochondrial apoptotic, and 6-shogaol decreased Bcl-2 expression while up-regulating Bax [111]. Down-regulation of MRP-1 expression by 6-shogaol in cancer cells has also been strongly confirmed in reports of liver cancer. 6-shogaol combined with 5-fluorouracil (5-FU) triggers apoptosis and cell cycle arrest in hepatocellular carcinoma cells by inhibiting AKT/mTOR/MRP-1 signaling. In the cancer cells treated with 6-shogaol-5-FU, the expression levels of cycle-related proteins were. Reduced and the expression of MRP-1 was down-regulated, which inhibited cell viability, promoted G0/G1 cell cycle arrest, and accelerated apoptosis. Furthermore, down-regulation of MRP-1 mRNA by 6-shogaol was associated with AKT inactivation, and AKT activation or MRP1 upregulation reversed these effects of combination therapy on cancer cells [61].

### Oxaliplatin、fluorouracil and irinotecan

Oxaliplatin, fluorouracil (FU) and irinotecan are commonly used in the treatment of colorectal cancer (CRC), but their anticancer effects are limited by severe chemoresistance. Marta et al. explored the efficacy of 6-shogaol in combination with these chemotherapeutic agents in the treatment of colorectal cancer. 6-Shogaol, FU, FOLFIRI (FU + irinotecan), FOLFOX (FU + oxaliplatin), and FOLFOXIRI (FU + oxaliplatin + irinotecan) were all effective in reducing SW480 or SW620 cell viability, ranging from 30 to 65%. However, 6-shogaol treatment of cancer cells in combination with chemotherapeutic agents resulted in a significant increase in cytotoxicity (98%) as measured by viability. In addition, a hypoxic, glucose-deprived tumour microenvironment reduces the therapeutic effect of chemotherapeutic agents. In the simulated tumor microenvironment, 6-shogaol still inhibited the viability of SW480 and SW620 cells, while chemotherapeutic drugs showed no or very weak cytotoxicity against cancer cells in the tumor microenvironment. However, the addition of 6-shogaol to all combinations of these chemotherapeutic agents (FU, oxaliplatin, irinotecan) increases their toxicity to cancer cells in the tumor microenvironment [30, 112].

In addition to enhancing the anticancer effect, the combination of 6-shogaol and olisaplatin also eliminates or ameliorates the toxic side effects associated with the treatment of CRC with olisaplatin. As a third generation platinum-based chemotherapeutic agent, the toxic effects of olisaplatin are significantly reduced, but it can still cause other side effects such as fatigue, nausea, vomiting and neuropathic pain. One of these, olisaplatin-induced peripheral neuropathic headache, has become a severe factor in interrupting treatment. A recent study showed that 6-shogaol administered intraperitoneally to mice at a dose of 10 mg/kg had an analgesic effect on oryzaplatin-induced neuropathic pain.This analgesic effect produced by 6-shogaol is associated with the release of GABA neurotransmitters mediated by 5-HT1 and 5-HT3 receptors in the spinal cord. GABA is an inhibitory neurotransmitter in the central nervous system. Elevated concentrations of GABA inhibit pain, whereas reduced concentrations can induce neuropathic pain [113]. These studies have shown that 6-shogaol not only has good anti-cancer potential on its own but also has the advantage of being used in combination with chemotherapeutic agents to increase efficiency and reduce toxicity.

### Gefitinib

Gefitinib, also known as Iressa, is an epidermal growth factor receptor (EGFR) tyrosine kinase inhibitor and a potential clinical anticancer drug, particularly with significant anti-tumor activity against non-small cell lung cancer (NSCLC) and ovarian cancer [114]. However, the chemical resistance induced by gefitinib in the treatment of various types of cancer is an unavoidable disadvantage. In a recent study, it was found that the combination of 6-shogaol and gefitinib can effectively overcome gefitinib mediated resistance. This synergistic effect is achieved by inhibiting the EMT process and activating ER stress in gefitinib resistant ovarian cancer cells with 6-shogaol. Among them, 6-shogaol + gefitinib upregulates E-cadherin and downregulates N-cadherin, vimentin, slugs, and snails are important reasons for overcoming gefitinib mediated resistance [115].

### Methotrexate

Methotrexate (MTX) is a well-known chemotherapy drug, and its anti-tumor mechanism is through inhibiting purine synthesis, inhibiting the S-phase of the cell cycle, and ultimately triggering cell apoptosis [116]. Although MTX has significant effects in the treatment of leukemia, breast cancer, head and neck cancer and other cancer types, it has a wide range of side effects, including bone marrow suppression, immunosuppression, mucositis, hepatotoxicity, alopecia, nausea and vomiting [117]. The combination of 6-shogaol and MTX not only enhances its therapeutic effect but also reduces its adverse reactions during the anti leukaemia process. The combination therapy of 6-shogaol and MTX showed synergistic cytotoxic effects on Nalm-6 cell line and patient primary cells. By using the MTT assay to calculate the viability of tumor cells, it was found that compared to using MTX alone, 6-shogaol/MTX had significantly higher growth inhibition effects, accounting for 36.54% and 51.93%, respectively. This combination therapy greatly improves the quality of life of patients during chemotherapy and reduces the recurrence rate [70].

## 6-Shogaol potential drug delivery

6-Shogaol has various pharmacological activities and abundant natural resources, especially in showing strong anticancer activity among numerous tumor types, with huge anticancer potential. Unfortunately, as 6-shogaol is a fat-soluble drug, its non-aqueous solvent form has relatively low bioavailability and high toxicity [118], Which limits its clinical application and development prospects. Therefore, finding a suitable drug delivery system is a successful strategy to solve this shortcoming. Nanodrug carriers have attracted the attention of researchers due to their excellent properties, such as improving drug bioavailability, enhancing drug targeting, and reducing drug toxicity and side effects [119]. At present, nanocarriers and cyclodextrin inclusion complexes are the two main delivery systems for 6-shogaol to solve the problems of low solubility and low bioavailability including nanoparticles [120–125], ultrasonic nanobubble carriers [118], micelles [126], solid lipid nanoparticles [127], Self-Microemulsifying [128], β-Cyclodextrin inclusion complex and Hydroxypropyl-β -cyclodextrin inclusion complex [129].

### Nanocarriers

#### Nanoparticles

Nanoparticles, due to their good biocompatibility and biodegradability, make them one of the successful strategies to increase the solubility and bioavailability of 6-shogaol. Zhang et al. used a versatile single-step surface-functionalising technique to prepare PLGA/PLA-PEG-FA nanoparticles loaded with the 6-shogaol [NPs-PEG-FA/6-shogaol]. NPs-PEG-FA/6-shogaol exhibited more potent anti-inflammatory effects than free 6-shogoal in dextran sodium sulfate (DSS)-induced colitis in mice [123]. Yang et al. used natural ginger lipids (NL) encapsulated with 6-shogaol (6-S-NL) in consideration of the safety of the drug delivery system and the reuse of medicinal resources. They orally delivered natural ginger lipid encapsulated 6-shogaol NPs to the large intestine and observed that their therapeutic effect against ulcerative colitis(UC) and bioavailability were superior to that of free 6-shogaol [121]. Subsequently, Yang et al. extracted total lipids from ginger-derived exosomal lipid nanoparticles (eLNPs) and use a thin-film hydration method to reassemble them into a natural LNP (nLNP) delivery system [120]. They loaded 6-shogaol into nLNP (6S/nLNP) to treat inflammatory bowel disease (IBD) by modulating the composition and function of the intestinal microbiota. Surprisingly, 6S/nLNP is five times more potent than free 6-shogaol in treating mice with inflammatory bowel disease. In addition, the investigators analyzed fecal metabolites from 6S/nLN-treated mice and found that these metabolites showed potent anti-inflammatory effects on LPS-induced inflammation and significantly reduced the expression of pro-inflammatory cytokines (TNF-α, IL-1β and IL-6) [120].

#### Ultrasound combined with NBs

Nanobubbles (NBs) are nano-sized carriers that are currently used as effective drug/gene delivery systems [130]. Ultrasonic transmission technology is a biophysical process based on the combination of ultrasound and NBs for cell pore formation, which is called ultrasonic cavitation [131]. In recent studies, it was reported that the combination of ultrasound and phase change nanobubble drug targeted delivery system significantly prolonged the blood circulation time of drug 6-shogaol, improved its pharmacokinetics and biological distribution [118]. The system consists of a liquid perfluorocarbon (PFC) core and a phospholipid shell that uses phospholipids as cofactors to deliver 6-shogaol to the tumor. Nanobubbles loaded with 6-shogaol combined with an ultrasound-targeted delivery system are effective and selective for epithelial ovarian cancer (EOC) [118].

#### Solid-lipid nanoparticles (SLNs)

Solid lipid nanoparticles (SLNs) have been regarded as promising nanocarriers to improve hydrophobic drugs' solubility and oral bioavailability [132, 133]. Wang et al. encapsulated 6-shogaol in solid lipid nanoparticles (SLN) for the first time, and the encapsulation efficiency reached 87.67%. Solid lipid nanoparticles (SSLNs) containing 6-shogaol are composed of a mixture of medium chain triglycerides and monostearate glycerides as the lipid core, a mixture of span 80 and Tween 80 as the emulsifier, and high-purity (98.65%) 6-shogaol as the therapeutic agent [127]. The obtained SSLNs are stable, uniform, and dispersed. Importantly, compared to free drugs, the in vitro release and in vivo oral bioavailability of SSLNs have been significantly improved [127].

#### Micelles

Micelles are the preferred targeted delivery system for many hydrophobic anticancer drugs [134]. Zang et al. loaded 6-shogaol into a novel micelle (SM), which increased its oral bioavailability by 3.2 times. It is worth noting that the in vitro cytotoxicity of SMs in HepG2 cells is significantly higher than that of free 6-shogaol [126]. The PEG derivative of linoleic acid (mPEG2K-LA) is used as a material for forming micelles to encapsulate 6-shogaol. The prepared 6-shogaol loaded micelles (SMs) increased the sensitivity of tumor cells to the prototype drug [126]. The in vitro anti-tumor activity of SMs showed that the cytotoxicity of SMs to HepG2 cells was significantly stronger than that of free 6-shogaol at low concentrations (5–50 μg/mL). Notably, the micellar material mPEG_2k_-LA showed little cytotoxicity to HepG2 even at high concentrations (1 mg/mL). Thus, it is suggested that the potency of SMs to increase cytotoxicity is attributed to the increased solubility of 6-shogaol by the micellar carrier and the improved bioavailability of the drug by avoiding passive diffusion through carrier-mediated endocytosis of the micelles into cells due to their ultra-small size [126].

#### Self- microemulsifying

The self-microemulsifying drug delivery system (SMEDDS) is a new type of drug carrier, which is a thermodynamic stable system composed of oil, water, surfactants or co solvents. The main driving force for its formation is ultra-low interfacial tension [135]. SMEDDS is considered the most promising carrier for insoluble drugs at present, as it has the advantages of promoting hydrophobic drug solubility, increasing drug stability, improving patient compliance, rapid onset, and easy preparation [128]. Recently, a study on the loading of 6-shogaol on SMEDDS showed that 6-shogaol can form spherical and uniform droplets under the conditions of 18.62% W/W ethyl oleate (oil phase) and Tween 80 (surfactant) to PEG 400 (cosurfactant) (1.73:1, W/W) ratio, with an average particle size and polydispersity of 20.00 ± 0.26 nm and 0.18 ± 0.02. Compared with free 6-shogaol, the cumulative release of 6-shogaol-SMEDDS formulation significantly increased, and more importantly, the relative oral bioavailability of the drug increased. Histological studies have confirmed that oral administration of 6-shogaol-SMEDDS formulations in hyperuricemia rats has better therapeutic effects than free drugs, and SMEDDS enhances the therapeutic efficacy of 6-shogaol [136].

### Cyclodextrin and its derivatives

Cyclodextrins (CDs) are cyclic oligosaccharides that have a high affinity for forming inclusion complexes with hydrophobic drugs, thereby altering the properties of the target drug, including enhancing its solubility, physical and chemical stability. They are commonly used to enhance the solubility and bioavailability of hydrophobic drugs [137]. Inclusion of 6-shogaol with β-cyclodextrin (β-CD) by the saturated aqueous solution method to form an inclusion complex of 6-shogaol/β-CDs (6-S-β-CDs), which showed significantly higher in vitro release, intestinal absorption and oral bioavailability compared to free 6-shogaol [129]. Hydroxypropyl-β-cyclodextrin (HPβCD) is a derivative of β-CD, which shows greater affinity for 6-shogaol encapsulation than β-CD, and cyclodextrin derivatives are more effective in complexing to promote the formation of inclusion complexes, which in turn increases the solubility of drug molecules. In addition, the hot melt extrusion (HME) processing method can not only more efficiently promote the encapsulation of 6-shogaol and cyclodextrin derivatives, but also enhance the anti-inflammatory activity of its complex in vivo [138].

## Conclusion

In a word, 6-shogaol, as the active ingredient in ginger, is proven to be a very potential natural anti-cancer product. It shows good therapeutic prospects in many cancer types. In addition, it is more interesting that the sensitivity of 6-shogaol to cancer and non-cancer cells varies greatly. Generally, 6-shogaol has no or almost no toxicity to normal cells at the lethal dose of cancer cells. This characteristic of 6-shogaol is in line with the concept of pursuing the development of effective and low-toxicity anti-cancer drugs to improve the quality of life of cancer patients during treatment. This paper comprehensively summarises the anti-cancer characteristics, anti-cancer mechanism, synergistic effect and potential delivery system of 6-shogaol. In addition, the molecular targets and pathways involved in 6-shogaol anti-cancer were emphasized. It is hoped to provide a theoretical basis and research ideas for more in-depth and comprehensive anti-cancer research of 6-shogaol in the future and have certain guiding significance for the future clinical development of 6-shogaol.

## Future outlook

6-shogaol is the main active ingredient in Chinese herbal medicine ginger, and its anticancer effect in various types of cancer has been confirmed in many studies. Currently, the research fervour on 6-shogaol is focused on the study of the anti-cancer mechanism of 6-shogaol, so there is a clearer understanding of the anti-tumour mechanism of 6-shogaol and the molecular signaling pathways involved. However, it is worth noting that a small number of studies have found that metabolites of 6-shogaol in mice (e.g. M2 and M13) exhibit stronger anti-inflammatory and anti-cancer activity than 6-shogaol itself. However, this new finding does not seem to have attracted much attention from researchers, and the reasons for the enhanced activity of these metabolites have not been explored further. Therefore, future research could be appropriately inclined to investigate the structures of these metabolites with more excellent anticancer activity, identify the structural differences between these metabolites and 6-shogaol, and then confer the new structures present in these metabolites of 6-shogaol by means of structural modifications. In addition, structurally modified 6-shogaol derivatives can be subjected to in vitro and in vivo antitumour activity studies to test whether structurally modified 6-shogaol exerts a more potent toxic effect on tumor cells. In addition, more studies on the metabolism of 6-shogaol through different routes of administration could be attempted to identify the most useful routes of administration for different types of cancer and to prepare more adequately for the transition from preclinical to clinical studies of 6-shogaol.

## Data Availability

Not applicable.

## Abbreviations

*Zingiber officinale Roscoe*: Ginger
PAPR: Poly-ADP-ribose polymerase
ER: Endoplasmic reticulum
IRE1: Inositol requiring enzyme-1
PERK: PKR-like ER-related kinase
ATF6: Transcription factor 6
CHOP: C/EBP-homologous protein
ROS: Reactive oxygen species
NACL: N-acetylcysteine
MMP: Matrix metalloproteinases
ECM: Extracellular matrix
EMT: Epithelial-mesenchymal transition
VEGF: Vascular endothelial growth factor
uPA: Urokinase-type fibrinogen activator
GSH: Reduced glutathione
STAT3: Signal transducer and activator of transcription 3
CCL2: CC-chemokine ligand 2
NF-kB: Nuclear factor kB
DSS: Dextran sodium sulfate
Lcn-2: Lipocalin-2
UC: Ulcerative colitis
CRC: Colorectal cancer
IL-6: Interleukin-6
IL-8: Interleukin-8
iNOS: Inducible nitric oxide synthase
HO-1: Heme oxygenase-1
MDRs: Multidrug resistance transporters
β-CD: β-Cyclodextrin
MTX: Methotrexate
